# Modeling the Spatial Distribution of Chronic Tumor Hypoxia: Implications for Experimental and Clinical Studies

**DOI:** 10.1155/2012/410602

**Published:** 2012-01-29

**Authors:** Gibin Powathil, Mohammad Kohandel, Michael Milosevic, Siv Sivaloganathan

**Affiliations:** ^1^Department of Applied Mathematics, University of Waterloo, Waterloo, ON, Canada N2L 3G1; ^2^Center for Mathematical Medicine, Fields Institute for Research in Mathematical Sciences, Toronto, ON, Canada M5T 3J1; ^3^Radiation Medicine Program, Princess Margaret Hospital; Department of Radiation Oncology, University of Toronto, Toronto, ON, Canada M5G 2M9

## Abstract

Tumor oxygenation status is considered one of the important prognostic markers in cancer since it strongly influences the response of cancer cells to various treatments; in particular, to radiation therapy. Thus, a proper and accurate assessment of tumor oxygen distribution before the treatment may highly affect the outcome of the treatment. The heterogeneous nature of tumor hypoxia, mainly influenced by the complex tumor microenvironment, often makes its quantification very difficult. The usual methods used to measure tumor hypoxia are biomarkers and the polarographic needle electrode. Although these techniques may provide an acceptable assessment of hypoxia, they are invasive and may not always give a spatial distribution of hypoxia, which is very useful for treatment planning. An alternative method to quantify the tumor hypoxia is to use theoretical simulations with the knowledge of tumor vasculature. The purpose of this paper is to model tumor hypoxia using a known spatial distribution of tumor vasculature obtained from image data, to analyze the accuracy of polarographic needle electrode measurements in quantifying hypoxia, to quantify the optimum number of measurements required to satisfactorily evaluate the tumor oxygenation status, and to study the effects of hypoxia on radiation response. Our results indicate that the model successfully generated an accurate oxygenation map for tumor cross-sections with known vascular distribution. The method developed here provides a way to estimate tumor hypoxia and provides guidance in planning accurate and effective therapeutic strategies and invasive estimation techniques. Our results agree with the previous findings that the needle electrode technique gives a good estimate of tumor hypoxia if the sampling is done in a uniform way with 5-6 tracks of 20–30 measurements each. Moreover, the analysis indicates that the accurate measurement of oxygen profile can be very useful in determining right radiation doses to the patients.

## 1. Introduction

Hypoxia is a feature of many solid malignant tumors and influences malignant disease progression, development of metastases, clinical behavior, and response to conventional treatments like radiotherapy [1–5]. Hypoxia may broadly be thought of as either acute, due to microregional fluctuations in blood flow over minutes to hours, or chronic, caused by abnormal vascular architecture with long intravascular transit times and long distances for oxygen diffusion through the tumor interstitium [3–5]. A proper assessment of the distribution of tumor hypoxia at initial presentation could aid in the design of appropriate therapeutic approaches for individual patients, thereby improving control rates and survival while reducing side effects [6–8].

Several approaches are commonly used to measure hypoxia in patient and experimental tumors, including polarographic electrode techniques and nitroimidazole binding as determined by flow cytometry, immunohistochemistry or PET imaging [4, 9–14]. An alternative approach that has not been as extensively studied uses theoretical simulations derived from mathematical models of oxygen transport phenomenon tailored to individual tumor characteristics such as blood vessel distribution. Previous theoretical investigations have shown that microvascular heterogeneity can substantially affect the distribution of hypoxia [15, 16]. Dasu et al. [16] developed a coarse-grain model of vascular networks as part of a more general theoretical model of tumor oxygenation; the authors analyzed different oxygenation dynamics based on a lognormal distribution of intervascular distances and studied their relationships to different hypoxic conditions. Based on experimentally derived data and numerical simulations, Secomb et al. [17] showed that O_2_ consumption is the most important factor influencing the local *p*O_2_ distribution in the tumors. Kohandel et al. [18] developed a mathematical model that incorporated tumor cells, vascular network, oxygen concentration, and treatment effects and used it to study the optimal combination of antiangiogenic and radiotherapy treatments. A random initial distribution of perfused vessels was used in this study to simulate oxygen distribution. However, it may be of greater biologica and clinical relevance to use tumor-specific microvascular distributions derived from biopsies.

 Here, we introduce a theoretical approach to model tumor hypoxia using known spatial distributions of the perfused tumor vasculature obtained from histological sections. We simulate oxygen distributions and calculate hypoxic fraction in two ways corresponding to sampling with a polarographic electrode and binding of a nitroimidazole agent. We further demonstrate that the simulated results correlate well with hypoxia measured directly in the same tumor sections [19]. The simulated oxygen distribution is then used to evaluate the radiation response under various hypoxic fractions.

## 2. Mathematical Model and Methods

### 2.1. Computational Domain

**Figure 1:**
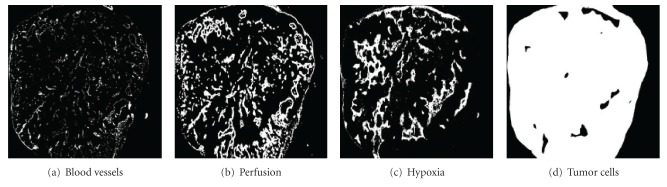
Binary images of one of the eight glioma xenographt cross-sections, illustrating tumor blood vessels, perfused vessels, hypoxic area, and total tumor area, respectively.

Representative, high-resolution, two-dimensional, histologic images of eight human glioma xenografts were gratefully received from P. F. J. W. Rijken, Department of Radiotherapy, University of Nijmegen, The Netherlands. An immunofluorescence staining technique was used to assess vascularity, perfusion, and hypoxia using 9F1 (mouse-specific endothelial marker), Hoechst 33342, and either pimonidazole or a similar agent 7-[49-(2-nitroimidazol-l-yl)-butyl]-theophylline (NITP), as described previously [19]. The stained tumor sections were than scanned and a threshold was applied to generate binary masks for each of the three parameters as illustrated in Figure 1 for a representative tumor. These binary images were used as computational domains on which a system of equations governing tumor growth and oxygen distribution was solved.

 Here, the perfused vasculature is considered to be the source of oxygen supply and thus gives the initial spatial distribution of oxygen concentration. This perfused vascular network (at a fixed point in time) is obtained by combining the images of perfused areas (Figure 1(b)) and vascular structures (Figure 1(a)) using the logical “AND” operation [19]. The images of hypoxic regions (Figure 1(c)) are later compared with the simulated hypoxic area to validate the mathematical model. The total tumor area is estimated from the binary image representing the tumor mask (Figure 1(d)).

### 2.2. Mathematical Model

Following Kohandel et al. [18], we use a simple reaction diffusion equation to represent the spatial and temporal changes in the oxygen and tumor cell concentrations. If *K*(*x*, *t*)  denotes the oxygen concentration at position *x* and time *t*, then its rate of change can be expressed as

(1) $$\begin{array}{cc} \frac{\partial K\left(x,t\right)}{\partial t} & =\nabla \cdot \left(D_{K}\left(x\right)\nabla K\left(x,t\right)\right)+\text{r}{\text{m}}_{p}\left(x,t\right)\\ & \;-\eta K\left(x,t\right)-\phi c\left(x,t\right)K\left(x,t\right), \end{array}$$

where *D*_*K*_ is the diffusion coefficient (considered to be a constant), *ϕ* is the rate of oxygen consumption by cells, and *η* denotes the rate of decay (assumed to be zero in the numerical simulations). Here, *m*_*p*_(*x*, *t*) stands for the density of perfused vessels (*m*_*p*_ *= *1 for the presence of blood vessel, and zero otherwise); thus the term *rm*_*p*_(*x*, *t*) describes the production of oxygen at rate *r*. The formulation of the model is then completed by prescribing no-flux boundary conditions and an initial condition (the initial spatial distribution of oxygen as determined from the image of perfused vasculature where the assumed intravascular oxygen distribution was prescribed only within this perfused vascular networks). Here, since we seek an instantaneous oxygen distribution map for a given vascular distribution, we do not consider the time evolution of the equations but rather use a computational time to iteratively reach a steady-state-like condition (details follow).

Similarly, the temporal rate of change of cell concentration is considered as a net result of diffusion and proliferation. If we denote by *c*(*x*, *t*) the density of the cells at position *x* at time *t*, then

(2) $$\begin{array}{cc} \frac{\partial c\left(x,t\right)}{\partial t} & =\nabla \cdot \left[D_{c}\nabla c\left(x,t\right)\right]+\rho c\left(1-\frac{c}{c_{\lim}}\right)\\ & \;+\gamma m_{p}\left(x,t\right)c\left(x,t\right). \end{array}$$

Here, *D*_*c*_ is the diffusion coefficient (constant) of tumor cells, *ρ* is the proliferation rate, and *c*_lim⁡_ is the carrying capacity. The third term denotes the effect of the vascular network on the growth of cells [18]. As above, we use a no-flux boundary condition and an initial condition *c*(*x*, 0) = *c*_0_(*x*), where *c*_0_(*x*) defines the initial spatial distribution of tumor cells (assumed to be a Gaussian distribution). The parameter values are given in Table 1.

The linear quadratic (LQ) model is the most commonly used approach for studying the survival response of tumor cells to radiotherapy and the concomitant clinical results [21]. In the LQ model, the survival fraction of cells after a single radiation dose of *D* (Gy) is given by

(3) $$\begin{array}{c} S=\exp\left(-\alpha D-\beta D^{2}\right), \end{array}$$

where **α** and *β* are the radiosensitivity parameters. The chosen set of parameters (*α* = 0.3 Gy^−1^ and *β* = 0.03 Gy^−2^) gives a survival fraction of 48% at a dose *D* = 2 Gy, under well-oxygenated (normoxic) conditions. However, this radio sensitivity may vary based on the oxygenation status of the cell, in which hypoxic cells are considered to be more resistant to radiation [1]. This effect of various oxygen levels on the radiosensitivity can be quantified in an LQ model using the concepts of “oxygen enhancement ratio (OER)” or “oxygen modification factor (OMF)” [22–25], defined as

(4) $$\text{OMF}=\frac{\text{OER}\left(p{\text{O}}_{2}\right)}{\text{OE}{\text{R}}_{m}}=\frac{1}{\text{OE}{\text{R}}_{m}}\frac{\text{OE}{\text{R}}_{m}\cdot p{\text{O}}_{2}\left(x\right)+K_{m}}{p{\text{O}}_{2}\left(x\right)+K_{m}},$$

where *p*O*_2_*(*x*) is the oxygen concentration at position *x*, OER*_m_* = 3 (the maximum value under well-oxygenated condition), and *K*_*m*_ = 3 mm Hg (the *p*O_2_ at which half the maximum ratio is achieved) [25]. Consequently, the LQ model can be modified, as below, by incorporating the effects of the oxygen distributions:

(5) $$\begin{array}{c} S_{\text{ox}}=\exp\left[-\alpha \cdot \text{OMF}\cdot D-\beta {\left(\text{OMF}\cdot D\right)}^{2}\right]. \end{array}$$

In general, the OER can be also a function of radiation dose, and some studies have suggested that the maximal oxygen enhancement varies in the range of 2.5 to 3 with differences in radiation dosage [26–28]. This can be simply included into the revised LQ model by considering different OERs for the radiosensitivity parameters **α** and *β*, that is, OER_*α*_ and OER_*β*_. However, since we consider the normalized OER (OMF), the introduction of these separate functions will not produce a significant difference in the final survival fraction. Thus, we assume OER_*α*_ = OER_*β*_ in our simulations.

Here, we use this revised LQ model to study effects of heterogeneous oxygen distribution on the predicted survival rates after radiation therapy. To this end, we calculate the cell survival fraction while varying the dosage *D* for different oxygen profiles. Comparisons are made for six different cases: (a) entire tumor is normoxic (*p*O_2_ = 60 mm Hg), (b) entire tumor is anoxic (*p*O_2_ = 0 mm Hg), (c) entire tumor is moderately hypoxic (*p*O_2_ = 5 mm Hg), (d) a two-fold profile, either hypoxic or normoxic at each grid point (*p*O_2_ ≤ 5 mm Hg and *p*O_2_ > 5 mm Hg), (e) a histogram of oxygen distribution with bin width of 5 mm Hg (5(*i* − 1) ≤ *p*O_2_ ≤ 5*i*, *i* = 1,2, 3,…, 20), see Figure 8(a), and (f) full heterogeneous oxygen distribution (simulated value of *p*O_2_ at each grid point). For cases (d)–(f), where the oxygen distribution is not uniform, we calculate the final survival fraction by taking the weighted average (with  *w*_*i*,*j*_ = 1) of the survival fractions at each compartment or grid points [24]:

(6) $$\begin{array}{c} \text{S}{\text{F}}_{\text{ox}}=\frac{\sum_{i,j}w_{i,j}S_{\text{ox}}\left(p{\text{O}}_{\text{2}}\left(i,j\right)\right)}{\sum_{i,j}w_{i,j}}. \end{array}$$

### 2.3. Computational Approaches for the Quantification of Hypoxic Area

The spatial distribution of hypoxia in each tumor section was simulated using the mathematical model (6 mm square computational domain with 100 × 100 grid points using a finite difference method) and then quantitated using two approaches corresponding to techniques commonly used in the laboratory and clinic. First, the percentages of the total tumor areas with *p*O_2_ threshold less than values 2.5%, 5%, and 10% were calculated, simulating image analysis of a hypoxic marker. Second, *p*O_2_ was sampled along linear measurement tracks and the percentage of values less than these thresholds again calculated to simulate polarographic needle electrode measurements. The spatial distribution of hypoxia and the summary measures derived from each of these approaches were compared to the known distribution of hypoxia and hypoxic fraction from the nitroimidazole analysis (Figure 1(c)) in the same tumors. Here, the spatial distribution of the predicated hypoxia is analyzed by comparing the pixel-by-pixel values of the original binary images of hypoxia with the simulated ones.

The focus of this work is to simulate the spatial distribution of hypoxia at a snapshot in time that will result from a particular distribution of perfused vessels and intravascular O_2_ concentrations, rather than tracking the time evolution of hypoxia. Yet it takes some computational “time” to arrive at this snapshot from our initial domain (the computation begins on a domain in which only the vasculature has nonzero oxygen concentration). The absolute oxygen distribution evolves as computational time proceeds. Therefore, to avoid dependence of our hypoxia quantification on computational time, we require a definition of hypoxia that considers relative, rather than absolute, quantities. Since the blood vessels act as constant source of oxygen, we assume for computational convenience that at any time *t*, maximum oxygen concentration among all grid points represents the hundred-percent oxic condition in that tumor microenvironment. We define a square grid area as hypoxic (HP2.5) if the average oxygen concentration is below 2.5% of the maximum concentration (max⁡_*x*_(*K*(*x*, *t*))(2.5/100)). Similar definitions hold for the other hypoxia thresholds (HP5 and HP10). This approach yields a consistent hypoxic fraction at any computational time once the oxygen concentration in the model is reasonably diffused. In other words, this quantity achieves a steady-state condition for oxygen concentration, which is what we require since we estimate hypoxia according to a fixed spatial distribution of vasculature.

**Figure 2:**
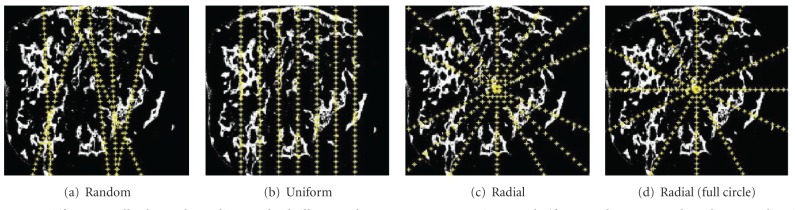
Different needle electrode reading methods illustrated over one representative sample (for a random approach, only one realization is shown).

Polarographic needle electrode measurements of hypoxia were simulated as linear tracks through the tumor. Four sampling patterns were used as illustrated in Figure 2: random, parallel, half radial (clockwise from 10–2 o'clock representing the situation where the tumor is accessible from only one side), and full radial (circumferential radial sampling). Measurements were taken at approximately 0.2 mm intervals along each track and 20–30 measurements were made per track to roughly match the total number of measurements used to estimate the hypoxia in previous laboratory and clinical studies [29]. Each individual measurement was assumed to correspond to the average oxygen concentration in a volume of tissue extending up to 60 *μ*m from the tip of the electrode, corresponding to the size of the computational grid [30]. Therefore, for simplicity, we assume here that (i) each electrode measuring point in the computational domain represents a group of cells (5 to 6 cells each with a diameter of 10–12 *μ*m in 2D) rather than a single cell and (ii) the oxygen concentration at the point of measurement represents the average oxygenation of a group of cells constituting that point. For all simulations, the percentages of oxygen readings less than 2.5%, 5%, and 10% were calculated using all measurements from all tracks, to yield estimates of HP2.5, HP5, and HP10, respectively.

### 2.4. Statistical Methods

The total variance in sampling the oxygenation status is the combined effect of within-tumor variance and between-tumor variance. Measurement of tumor *p*O_2_ is considered to be a predictive outcome assay only when the within-tumor *p*O_2_ variability is smaller than the variability among different tumors [31]. Using the estimated *p*O_2_ values from the electrode simulations, the variability of oxygenation status within and between tumor samples is estimated through variance components analysis by computing the ratio of within-tumor variance over the total variance for each reading method (uniform, random, and radial) [29]. This variance analysis is repeated for each additional track to compare the effects of the number of tracks on *p*O_2_ estimates and thus to obtain an optimum number of needle probes. To study the percentage of variation in evaluating the hypoxic proportions, the variance analysis is also performed using the two different approaches to quantify hypoxia (simulated percentage hypoxic area and needle electrode measurements):

(7) $$\begin{array}{cc} & \underset{\left(\text{Needle}\;\text{measurements}\right)}{\underset{︸}{\text{Percentage}\;\text{of}\;\text{total}\;\text{variance}}}\\ & \;=\frac{\mathrm{Var}\left(\text{Within}\;\text{tumor}\right)}{\mathrm{Var}\left(\text{Between}\;\text{tumors}\right)+\mathrm{Var}\left(\text{Within}\;\text{tumor}\right)}100, \end{array}$$

(8) $$\begin{array}{cc} & \underset{\text{Estimation}\;\text{Methods}}{\underset{︸}{\text{Percentage}\;\text{of}\;\text{totalvariance}}}\\ & \;=\frac{\mathrm{Var}\left(\text{Between}\;\text{methods}\right)}{\mathrm{Var}\left(\text{Between}\;\text{tumors}\right)+\mathrm{Var}\left(\text{Between}\;\text{methods}\right)}100. \end{array}$$

## 3. Results and Discussions

**Figure 3:**
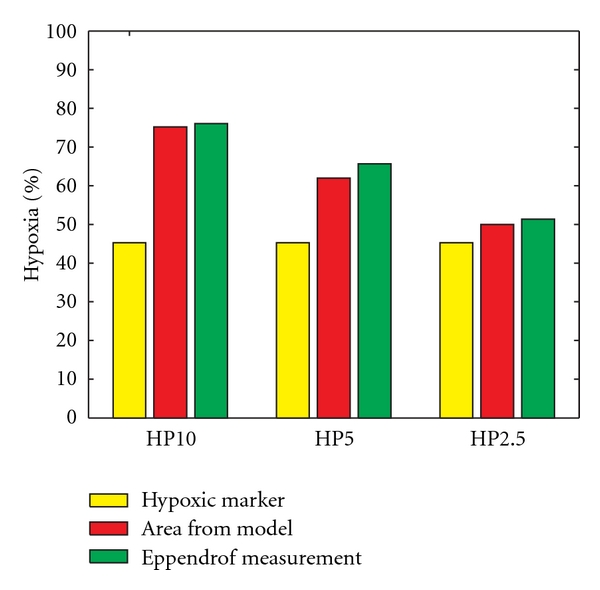
Comparison of hypoxic estimations at mild (HP10), moderate (HP5) and severe (HP2.5) hypoxic levels for a representative sample case.

The oxygenation status of a heterogeneous tumor is often quantified using polarographic electrode measurements or through nitroimidazole binding and biopsies. These invasive techniques have varying accuracy due to the restricted sampling space as well as limited accessibility. In this paper, we present an alternative theoretical approach that permits an exploration of the spectrum of hypoxic distributions that could possibly be associated with a particular vascular configuration. We used two-dimensional binary images of tumor cross-sections, with perfused tumor vasculature as a computational domain, on which a simple model for the oxygen distribution and tumor cell density was solved. The resulting hypoxic area was quantified through two different approaches and compared against the hypoxic proportions determined from the original biopsy images (Figure 3).

### 3.1. Hypoxic Area and Theoretical Needle Electrode Measurements

Herein, we have presented and utilized definitions of hypoxia corresponding to three different commonly considered threshold levels, that is, mild (HP10), moderate (HP5), and severe (HP2.5) hypoxic conditions. The percentage of total area that is hypoxic and the percentage of hypoxic readings (as determined by simulated needle electrode measurement) are then calculated with respect to these hypoxic thresholds. The results are compared against the known proportions estimated from the biopsy images (Figure 1(c)) to study the validity of the model as well as the accuracy of both the theoretical and the probing techniques. The HP10, HP5, and HP2.5 hypoxic fractions for one tumor cross-section are shown in Figure 3.

The hypoxic proportion, as estimated from the original image, is shown in yellow. The red and green boxes represent simulated hypoxia, which is quantified by estimating the percentage of total area that is hypoxic and through theoretical needle electrode measurements, respectively. In the case of HP2.5 (Figure 3), the proportion of model-generated hypoxia is in reasonable agreement with the proportion determined from the original images (a result that is consistent across the remaining samples of tumor cross-sections). It should be noted that the available binary image of hypoxic area (Figure 1(c)) allows us to estimate hypoxia only at a single-threshold level. Hence, hypoxic areas obtained from biomarker staining (yellow) are the same, and we do not expect to see agreement across all three-threshold levels in Figure 3.

**Figure 4:**
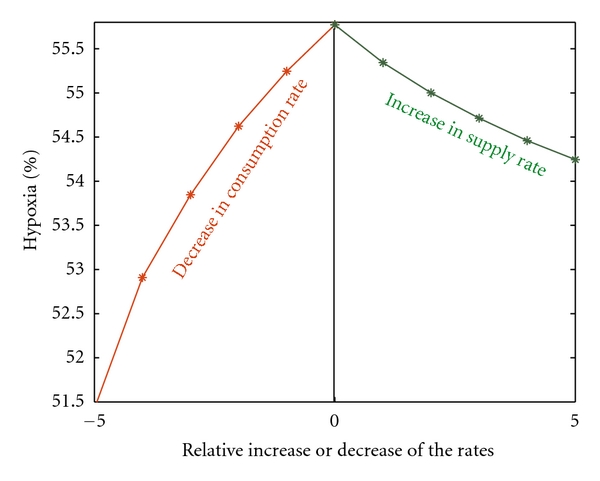
Change in hypoxia as a function of relative changes in production and consumption rates of oxygen. The figure indicates that a relative decrease in consumption might be an effective way to decrease the hypoxia.

 The binary images of hypoxic area obtained through biomarker staining reflect a number of factors related to tissue preparation, staining absorption, staining threshold, image acquisition, and image brightness. In several experimental studies [4, 11–13], it has been observed that intensity of hypoxic marker binding increases with increasing levels of hypoxia. According to the Raleigh et al. [12], pimonidazole bindings usually occur at levels less than 10 mm Hg, and half-maximal pimonidazole binding occurs around 2 mm Hg. Raleigh et al. [12] showed that HP10 measurements with *p*O_2_ needle electrodes correlate with pimonidazole binding surface area with a systematic offset of 36%, and this offset is smallest for HP2.5 (18%). Our analysis of simulated hypoxia using eight tumor samples (Figure 4) shows that a best overall agreement between the simulated and measured values is obtained at a threshold of 5% (HP5). Alternatively, a threshold of 2.5% (HP2.5) provides very good correlation with the measured values in four of the eight tumor samples and underestimates hypoxia in the other four. This difference between the simulated and experimental values may be either due to a component of superimposed acute hypoxia and/or to a higher rate of oxygen consumption than that used in the simulations. Moreover, the samples that underestimate hypoxic regime have a relatively higher vascular area as compared to the other four samples. This indicates the presence of acute hypoxia, which has not been included in the present model, as we do not have the access to the temporal images. Nevertheless, the spatial correlations between the simulated hypoxic distribution and the biopsy hypoxic area given in Table 2 indicate that these correlations fall within the range of 75 to 85%, showing that the presented model provides a satisfactory prediction of the spatial distribution of hypoxia.

Our definition of hypoxia also plays an important role in dictating the reliability of the estimates of hypoxic proportions obtained through computation. To test sensitivity of hypoxia estimates found using this definition with respect to changes in computational diffusion time, we analyzed HP10 values at different (nondimensional) time values and found that, for both theoretical measurement approaches, the hypoxic proportions estimated are similar for each time (result not shown). This supports the validity of our hypoxic definition, since a given tumor microenvironment with a fixed vascular network (fixed in the sense that we consider timescales too small to permit changes in perfused vascular geometry) should yield an approximately fixed hypoxic proportion over these small time intervals.

To study the relative sensitivity of various parameters involved in the present mathematical model, we have performed a comparative analysis of the variations in tissue hypoxia with respect to the relative changes in production/supply (increasing *r*) and consumption (decreasing *ϕ*) of oxygen concentration as illustrated in Figure 4. The analysis shows that the consumption of oxygen plays a vital role in defining local tissue oxygenation as compared to the oxygen supply and thus reducing the oxygen consumption rate may be more effective in lowering the hypoxic proportions within the tissue, which is consistent with previous studies [17]. Moreover, this uncertainty in the parameter values does not significantly influence the final simulation results of hypoxia (Figure 4).

**Figure 5:**
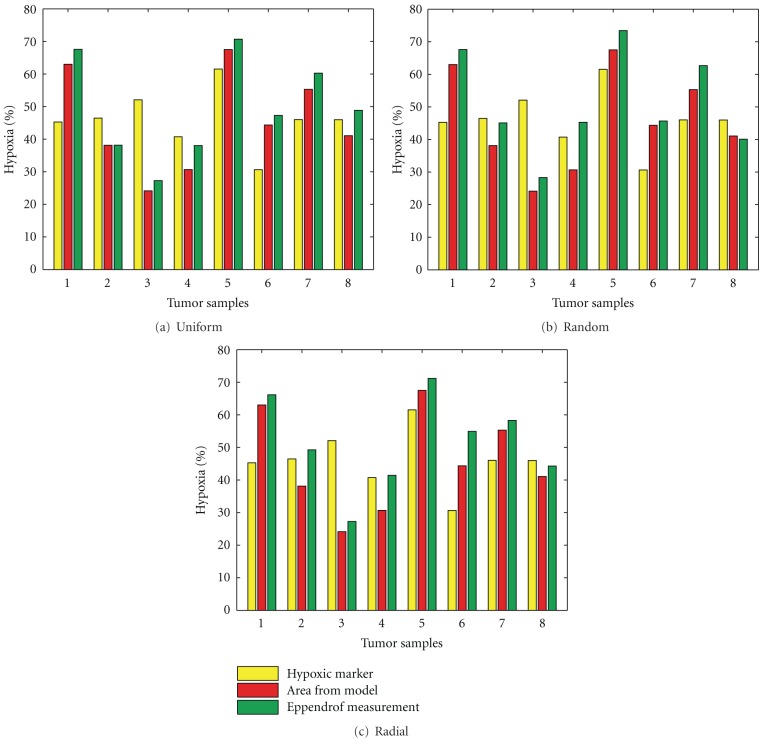
HP5 estimations for eight tumor cross-sections using three different needle measurement approaches. (Yellow: percentage of hypoxic area from the original image, red: percentage of hypoxic area from model, and green: HP5 estimation using needle electrode.)

Figure 5 illustrates model-obtained hypoxia estimates with the HP5 threshold for eight different tumor cross-sections, using three different methods of simulated needle electrode tracking (viz., the uniform, radial, and random methods, depicted in Figure 2). As can be seen from the graph, these three methods of needle tracking give similar results and all are in good agreement with the simulated estimates of hypoxia found by calculating the percentage of the total area that is hypoxic (red and green bars in Figure 5). This supports the general opinion that polarographic electrodes give reasonable estimates of tumor oxygen status, and in fact, several researchers consider this to be the “gold standard” method for characterizing hypoxia in human tumors [10, 32]. However, it should be noted that our simulated needle electrode measurements are not subject to instrumental error, which is inherent in practice.

### 3.2. Analysis of Variance

**Figure 6:**
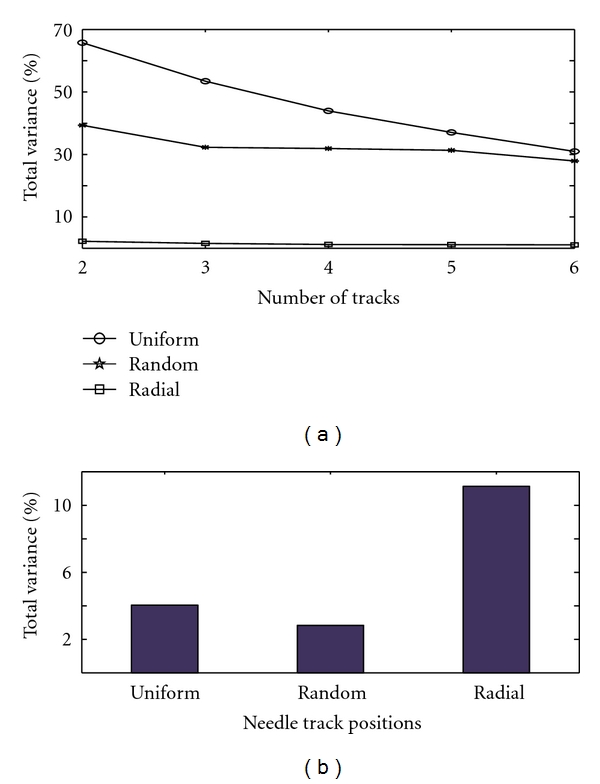
(a) The percentage of total variance due to within tumor variance (for the entire eight samples), as a function of number of tracks. The plot shows that 5-6 tracks of 20–30 measurements give an optimal reading of hypoxia. (b) The percentage of total variance due to the variance between two methods of hypoxia estimation for three different electrode measurements approaches (analysis of eight samples). This shows that radial sampling is less accurate than uniform and random sampling.

Here, we use statistical analysis with two purposes in mind: to consider the fraction of within-tumor variance (relative to total variance) associated with each pattern of needle insertion in an effort to predict an optimum number of tracks required for satisfactory measurement, and secondly, to determine the best tracking pattern by considering the fraction of variability between two different estimation methods among the tumor samples (relative to total variance). We note that the differences between these three different approaches to needle tracking are not clearly evident in Figure 6(a), and so we use variance components analysis (ANOVA) to compute the within- and between- tumor variability of needle electrode measurements.

Similar analyses comparing the variability of different oxygen measurements have been carried out in several experimental studies [29, 31, 33]. One may use this kind of analysis when the assumption that the error terms are normally distributed holds; hence, before using this method to estimate the variances, we analyzed our simulated data and found that the errors approximately follow a normal distribution. The variances were then calculated using statistical software, and variability was expressed in terms of percentage of total variance. Figure 6(a) shows this percentage of total variance as a function of number of needle tracks for the three different needle track arrangements.

The variance analysis results of Figure 6(a) show that the percentage of total variance due to the variance within the tumor is small for the random approach when compared to the radial and uniform approaches, whereas the uniform method of tracking has maximum contribution of within variance to the total variance. However, this analysis may not necessarily permit one to conclude that the radial approach is better than other methods to sample hypoxia but rather may just be representative of this tracking method producing less spatial variation between electrode tracks. Furthermore, these results also indicate that the percentage of total variance due to within-tumor variance is decreased with an increase in the number of needle tracks and that this decrease is minimal when the number of tracks is increased from five to six. Thus, the (minor) statistical benefits of increasing the number of tracks beyond this point are likely to be offset by the disadvantages of increased invasiveness. This indeed brings us to the same conclusion made by Wong et al. [29] for the case of cervical cancers that the use of five (20–30 measurements) tracks is optimal when sampling a cervix cancer for obtaining a reliable and reproducible oxygenation status.

**Figure 7:**
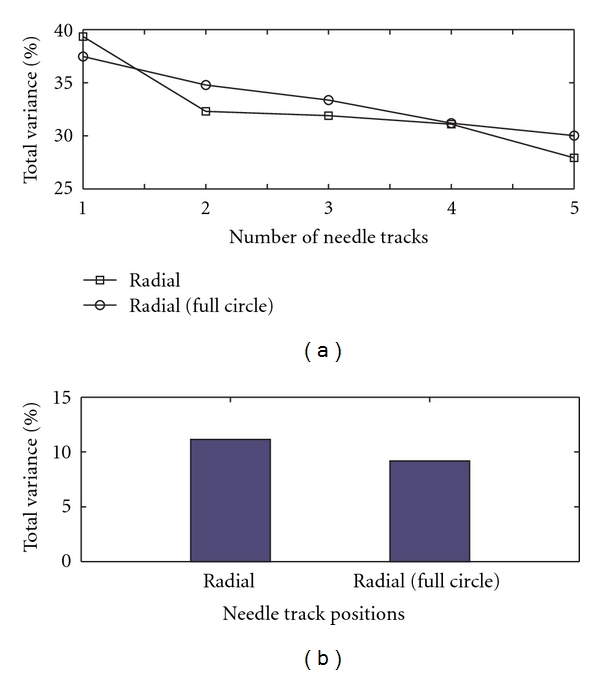
Plots showing the variance comparison between two different types of radial approaches (considering eight sample cases) to investigate the decreased accuracy of radial sampling.

The differences among the three different needle tracking approaches are further studied with variance analysis by calculating the percentage of total variance (between-estimation methods and between-tumor sections) due to the variations between two different estimation methods (i.e., by finding the hypoxic area and through the needle electrode method). This is repeated for all three sample electrode tracking approaches and the results are shown in Figure 6(b). It is clear from Figure 6(b) that the contribution of variations between the two different methods of quantifying hypoxia (specifically, the area approach and electrode sampling method) to the total percentage of variations is much higher in the case of the radial method than for the other tracking strategies. This implies that the radial method of electrode sampling is less accurate in sampling hypoxia than the other two approaches even though it has only small variations for within-tumor measurements (Figure 6(a)). This may be due to the manner in which we select the needle tracks in the radial position: here, we assume that the tumor is accessible only from one side of the sample (as would likely be the case* in situ*), reaching the whole tumor (Figure 2(c)), and so all six tracks are situated between the 10 o'clock and 2 o'clock positions. This dictates that the needle tracks be close to each other, resulting in a smaller effective sampling area which in turn makes the variations within the tumor smaller and the variation between the estimation methods higher. To verify this inference, we have introduced another theoretical tracking approach, for which we assume that the tumor is accessible from all the o'clock directions (Figure 2(d), from 9 to 3 o'clock positions, which we shall call radial (full circle), and compared this against the above results of the radial approach (Figure 7). It can be seen from the figure that when we increase the sampling area by spreading the tracks across a greater proportion of the “circle,” the percentage of total variance due to the variance of within-tumor measurements increased while the contribution of between-methods (estimation) variance to the percentage of total variance decreased—although it did not decrease to a value as low as that for the uniform approach. Hence, we may reasonably conclude that a uniform spacing of electrode tracks gives a good estimation of the hypoxic proportion when compared to the other methods.

### 3.3. Radiation Response

The oxygenation status of a tumor is generally considered to be an important intrinsic factor in determining radiation response, where viable hypoxic cells are more resistant to radiation than well-oxygenated cancer cells [1]. The hypoxia-dependent limitations of radiotherapy necessitate consideration of the spatial distribution of hypoxia within a tumor in order to estimate cancer cell survival fraction due to irradiation. Hence, we use the hypoxia distributions, discussed in the previous sections, and apply the modified LQ model to calculate the survival fraction for a single radiation dose of *D* (Gy).

**Figure 8:**
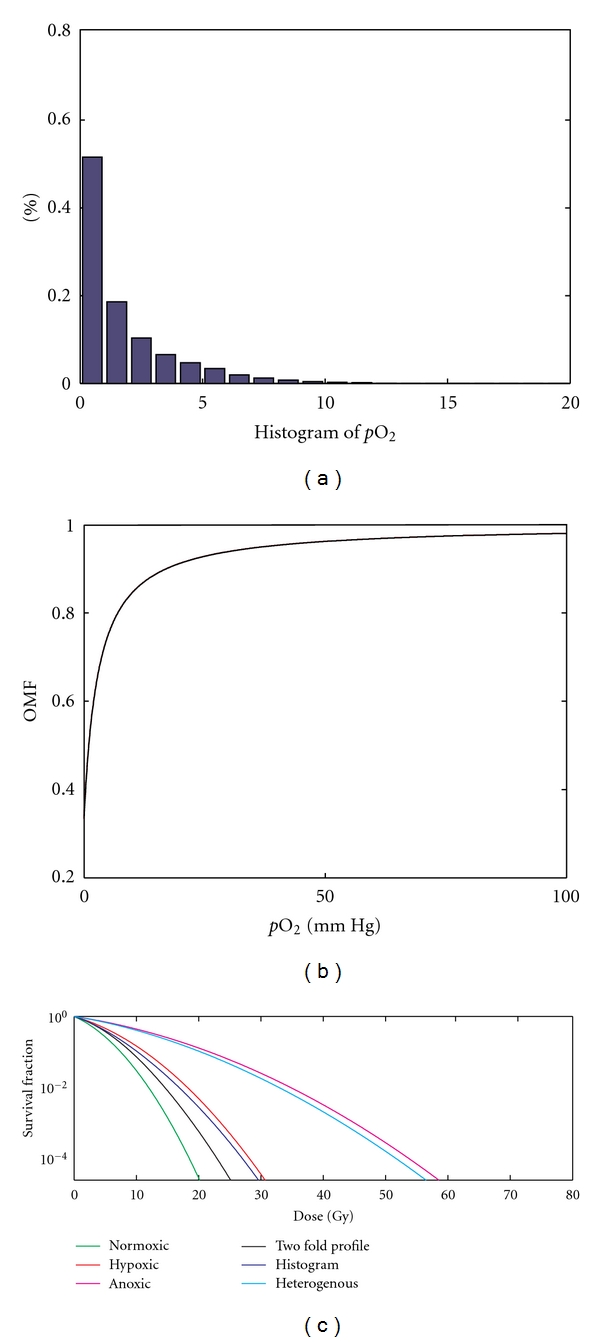
(a) Simulated oxygen distribution as histograms of width 5 mm Hg (for a representative case), (b) oxygen modification factor (OMR) as a function of the oxygen concentration, and (c) survival fraction for different cases of oxygen profiles considering the oxygen distribution of a representative case.

To study the effects of oxygenation status on tumor cell survival fraction, we considered six different cases of oxygen sensitivity profiles (listed in Section 2).  Figure 8 shows the model-derived oxygen distribution as histograms of width 5 mm Hg (Figure 8(a)), oxygen modification factor (OMF) as a function of the oxygen concentration (Figure 8(b)), and the survival fraction for various oxygenation profiles (Figure 8(c)). The results indicate that the oxygen concentration significantly affects OMF (Figure 8(b)); this is due to the fact that OMF increases very quickly (within 0–10 mm Hg) to its normalized value with increasing *p*O_2_ concentration. Hence, considering the sensitivity of the heterogeneous distribution of oxygen at each grid point, a much higher dosage is required to get the same survival fraction of cells compared to the other four cases (Figure 8(c)). This level of dosage is even higher than the case of a fully hypoxic tumor. However, this may be due to the assumption that the fully hypoxic tumor has a uniform oxygen distribution of 5 mm Hg (moderate hypoxia), while for the heterogeneous case most, of the cells have an oxygen concentration less than 5 mm Hg. Furthermore, this is clear from Figure 8(c) where the dosage level curve for heterogeneous distribution is almost coincident with the curve for complete anoxia (but lying slightly below). The reason for these similar results is due to the high sensitivity of severe hypoxic cells (cells with less than 5 mm Hg) with respect to radiation response, which is theoretically quantified using the OMF curve (Figure 8(b)). This OMF curve increases to its peak value with a relatively small increase in oxygen concentration (0–10 mm Hg), and hence cells with severe hypoxia give rise to similar survival effects as that of anoxic cells. The above results indicate the importance of the effects of oxygenation status in estimating the radiation response of tumor cells. Moreover, the accuracy of this estimation is closely dependent on detailed quantification of oxygen distribution inside the tumor rather than a classification into hypoxic or nonhypoxic compartments.

## 4. Conclusions

Tumor hypoxia is a common feature of advanced solid tumors wherein the metabolic demand for oxygen exceeds its supply or availability [1, 34]. Hypoxia occurs as a result of a stressful and abnormal vascular architecture in tumors, which itself arises mainly as a result of unregulated angiogenesis and thus contributes to the deficiency in oxygen delivery as well as to elevated interstitial fluid pressure [10, 34]. The lack of oxygenation of tumor cells can also be further exacerbated by the increase in diffusion distance and/or intervascular distance. Hence, a thorough knowledge and understanding of this complex microenvironment is a vital step in studying, estimating, and treating tumor hypoxia. There are several experimental methods, which can be used to describe the dynamics of these tumor vascular networks [19, 35]. Theoretical attempts have also been made to estimate tumor hypoxia by simulating a coarse-grain model for the tumor microenvironment [16].

The oxygenation status of a heterogeneous tumor is often quantified using polarographic electrode measurements or through nitroimidazole binding and biopsies. These invasive techniques often have varying accuracy due to the restricted sampling space as well as limited accessibility. In this paper, we present an alternative theoretical approach that might allow us to explore the spectrum of hypoxic distributions that could possibly be associated with a particular vascular configuration. Herein, we have used two-dimensional images of eight tumor cross-sections with perfused tumor vasculature as a computational domain on which a system of partial differential equations describing the distribution of hypoxia has been solved. As discussed in the previous sections, the resulting hypoxic area has then been quantified by two different approaches. To validate the findings from our mathematical model, these hypoxic estimates have been compared against the hypoxic proportions determined from the original images showing the hypoxic area according to pimonidazole binding.

In most tumors, the hypoxia that occurs is of mixed type [34] and since we are only considering the perfused vascular network as our source of oxygen supply, it may account for chronic as well as acute hypoxia at that specific point in time. However, the spatial distribution of perfused blood vessels may be unattainable through noninvasive techniques, whereas maps of the entire vascular network may, in the future, become available through modern high-resolution imaging techniques such as angiograms. In this event, the model can be used to generate hypothetical hypoxic conditions, by considering either the entire vascular network or by developing criteria for choosing the perfused vascular vessels from this complete network, which may constitute future work.

In conclusion, we have presented a simple diffusion model, which can satisfactorily estimate the oxygenation maps of a heterogeneous tumor with a given vascular network. We have shown that an estimate can be made of average tumor hypoxia that appears to be less sensitive to the characteristics of the vascular network as compared to the variations in O_2_ consumption. Thus, this approach can be used to quantify average tumor hypoxia knowing only the distribution of tumor vessels. Using this model, we have found that the polarographic electrode measurements accurately quantify the oxygenation status of the tumor microenvironment. Our studies show that five to six uniformly distributed equidistant measurement tracks with 20–30 measurements per track give the optimum balance between accuracy and invasiveness. The radiation response under various oxygenation conditions has also been analyzed using a simple model for the radiation effect and we have found that consideration of the heterogeneous distribution of oxygen plays an important role in the accurate prescription of radiation dosage. This type of theoretical study may be used to provide an alternative method of estimating hypoxia distribution in solid tumors, which may possibly help in the design of optimal, patient-specific, and accurate invasive estimation methods.

## Figures and Tables

**Table 1:** List of parameters.

Parameters	Symbol	Value	Reference
Diffusion constant for oxygen	*D*_*k*_	2.5 × 10^−5^ (cm^2^ s^−1^)	[20]
Rate of oxygen supply	*r*	8.2 × 10^−3^ (O_2_ s^−1^)	[20]
Cellular oxygen consumption*	*ϕ*	3.8 × 10^−13^ (cm^2^ O_2_ s^−1^ (cells)^−1^)	[15]
Diffusion constant for cells	*D*_*c*_	4.05 × 10^−9^ (cm^2^ s^−1^)	[18]
Proliferation rate	*ρ*	1.85 × 10^−6^ (s^−1^)	[18]
Carrying capacity	*c*_lim⁡_	2.1 × 10^11^ (cells s^−1^)	[18]
Cellular growth rate (effect of vasculature)	*γ*	2.96 × 10^−6^ (s^−1^)	[18]

*Assuming mass of 1 cell = 10^−9^ Kg.

**Table 2:** Spatial correlation (%) of hypoxic area.

Tumor sample	Percentage of correlations		
	HP2.5	HP5	HP10
1	77.6738	73.8076	68.3374
2	81.1242	79.7579	74.8348
3	80.701	83.1489	81.847
4	87.6884	85.6093	78.3471
5	73.3323	71.2458	68.0627
6	80.498	74.8546	66.3771
7	75.8521	71.6368	65.637
8	76.2209	73.0056	66.1667

## References

[1] Tannock IF, Hill RP, Bristow RG, Harrington L (2005). *The Basic Science of Oncology*.

[2] Bristow RG, Hill RP (2008). Hypoxia, DNA repair and genetic instability. *Nature Reviews Cancer*.

[3] Vaupel P (2008). Hypoxia and aggressive tumor phenotype: implications for therapy and prognosis. *The Oncologist*.

[4] Ljungkvist AS, Bussink J, Kaanders JHAM, Van Der Kogel AJ (2007). Dynamics of tumor hypoxia measured with bioreductive hypoxic cell markers. *Radiation Research*.

[5] Chaplin DJ, Olive PL, Durand RE (1987). Intermittent blood flow in a murine tumor: radiobiological effects. *Cancer Research*.

[6] Hockel M, Knoop C, Schlenger K, Vorndran B, Knapstein PG, Vaupel P (1994). Intratumoral pO2 histography as predictive assay in advanced cancer of the uterine cervix. *Advances in Experimental Medicine and Biology*.

[7] Nordsmark M, Overgaard M, Overgaard J (1996). Pretreatment oxygenation predicts radiation response in advanced squamous cell carcinoma of the head and neck. *Radiotherapy and Oncology*.

[8] Brizel DM, Sibley GS, Prosnitz LR, Scher RL, Dewhirst MW (1997). Tumor hypoxia adversely affects the prognosis of carcinoma of the head and neck. *International Journal of Radiation Oncology Biology Physics*.

[9] Trotter MJ, Chaplin DJ, Durand RE, Olive PL (1989). The use of fluorescent probes to identify regions of transient perfusion in murine tumors. *International Journal of Radiation Oncology Biology Physics*.

[10] Milosevic M, Fyles A, Hedley D, Hill R (2004). The human tumor microenvironment: invasive (needle) measurement of oxygen and interstitial fluid pressure. *Seminars in Radiation Oncology*.

[11] Kavanagh MC, Sun A, Hu Q, Hill RP (1996). Comparing techniques of measuring tumor hypoxia in different murine tumors: eppendorf _p_O_2_ histograph, [^3^H]misonidazole binding and paired survival assay. *Radiation Research*.

[12] Raleigh JA, Chou SC, Arteel GE, Horsman MR (1999). Comparisons among pimonidazole binding, oxygen electrode measurements, and radiation response in C3H mouse tumors. *Radiation Research*.

[13] Jenkins WT, Evans SM, Koch CJ (2000). Hypoxia and necrosis in rat 9L glioma and Morris 7777 hepatoma tumors: comparative measurements using EF5 binding and the Eppendorf needle electrode. *International Journal of Radiation Oncology Biology Physics*.

[14] Gerstner ER, Sorensen AG, Jain RK, Batchelor TT (2008). Advances in neuroimaging techniques for the evaluation of tumor growth, vascular permeability, and angiogenesis in gliomas. *Current Opinion in Neurology*.

[15] Secomb TW, Hsu R, Dewhirst MW, Klitzman B, Gross JF (1993). Analysis of oxygen transport to tumor tissue by microvascular networks. *International Journal of Radiation Oncology Biology Physics*.

[16] Dasu A, Toma-Dasu I, Karlsson M (2003). Theoretical simulation of tumour oxygenation and results from acute and chronic hypoxia. *Physics in Medicine and Biology*.

[17] Secomb TW, Hsu R, Ong ET, Gross JF, Dewhirst MW (1995). Analysis of the effects of oxygen supply and demand on hypoxic fraction in tumors. *Acta Oncologica*.

[18] Kohandel M, Kardar M, Milosevic M, Sivaloganathan S (2007). Dynamics of tumor growth and combination of anti-angiogenic and cytotoxic therapies. *Physics in Medicine and Biology*.

[19] Rijken PFJW, Bernsen HJJA, Peters JPW, Hodgkiss RJ, Raleigh JA, Van Der Kogel AJ (2000). Spatial relationship between hypoxia and the (perfused) vascular network in a human glioma xenograft: a quantitative multi-parameter analysis. *International Journal of Radiation Oncology Biology Physics*.

[20] Matzavinos A, Kao CY, Green JEF, Sutradhar A, Miller M, Friedman A (2009). Modeling oxygen transport in surgical tissue transfer. *Proceedings of the National Academy of Sciences of the United States of America*.

[21] Thames HD, Hendry JH (1987). *Fractionation in Radiotherapy*.

[22] Alper T, Howard-Flanders P (1956). Role of oxygen in modifying the radiosensitivity of E. Coli B. *Nature*.

[23] Wouters BG, Brown JM (1997). Cells at intermediate oxygen levels can be more important than the “hypoxic fraction” in determining tumor response to fractionated radiotherapy. *Radiation Research*.

[24] Dasu A, Toma-Dasu I, Karlsson M (2005). The effects of hypoxia on the theoretical modelling of tumour control probability. *Acta Oncologica*.

[25] Titz B, Jeraj R (2008). An imaging-based tumour growth and treatment response model: investigating the effect of tumour oxygenation on radiation therapy response. *Physics in Medicine and Biology*.

[26] Palcic B, Skarsgard LD (1984). Reduced oxygen enhancement ratio at low doses of ionizing radiation. *Radiation Research*.

[27] Skarsgard LD, Harrison I (1991). Dose dependence of the oxygen enhancement ratio (OER) in radiation inactivation of Chinese hamster V79-171 cells. *Radiation Research*.

[28] Freyer JP, Jarrett K, Carpenter S, Raju MR (1991). Oxygen enhancement ratio as a function of dose and cell cycle phase for radiation-resistant and sensitive CHO cells. *Radiation Research*.

[29] Wong RKW, Fyles A, Milosevic M, Pintilie M, Hill RP (1997). Heterogeneity of polarographic oxygen tension measurements in cervix cancer: an evaluation of within and between tumor variability, probe position, and track depth. *International Journal of Radiation Oncology Biology Physics*.

[30] Toma-Dasu I, Waites A, Daşu A, Denekamp J (2001). Theoretical simulation of oxygen tension measurement in tissues using a microelectrode: I. The response function of the electrode. *Physiological Measurement*.

[31] Brizel DM, Rosner GL, Prosnitz LR, Dewhirst MW (1995). Patterns and variability of tumor oxygenation in human soft tissue sarcomas, cervical carcinomas, and lymph node metastases. *International Journal of Radiation Oncology Biology Physics*.

[32] Olive PL, Banath JP, Aquino-Parsons C (2001). Measuring hypoxia in solid tumours: is there a gold standard?. *Acta Oncologica*.

[33] Nordsmark M, Loncaster J, Aquino-Parsons C (2003). Measurements of hypoxia using pimonidazole and polarographic oxygen-sensitive electrodes in human cervix carcinomas. *Radiotherapy and Oncology*.

[34] Vaupel P, Harrison L (2004). Tumor hypoxia: causative factors, compensatory mechanisms, and cellular response. *The Oncologist*.

[35] Bussink J, Kaanders JHAM, Rijken PFJW (1999). Vascular architecture and microenvironmental parameters in human squamous cell carcinoma xenografts: effects of carbogen and nicotinamide. *Radiotherapy and Oncology*.

